# The Process of Refining and Manufacturing Gold Foil

**Published:** 1899-05

**Authors:** John Hood


### THE DENTAL REGISTER.

VOL. LIII.] MAY, 1899. [No. 5.

### COMMUNICATIONS.

### The Process of Refining and Manufacturing Gold Foil.

BY JOHN HOOD.

Read before the Vermont State Dental Society.

We take gold in any form or quantity, from one to twenty-four karat, and melt it with silver, alloying it down to ten karat. It is then granulated into small shot by pouring into water when in a melted state. The granulated gold is then boiled in a retort on a sand bath in nitric acid until the silver and base metals are dissolved, the gold remaining in the shape of brown mud, which is then washed, dried and melted. The result is what we call parting gold, which is .996 or .998 fine, and it from this that most gold foils are manufactured. We then take this gold, roll it into a thin ribbon, immerse it in nitro-muriatic acid, which dissolves the gold and leaves the silver. Before, we dissolved the silver and left the gold.

This gold solution is then filtered to get rid of the chloride of silver, which is very light and floats to a certain extent. By filtering we get a pure solution. The gold is then precipitated.

After the gold is precipitated, it is first washed and dried, then melted and poured into ingots one inch wide, which are rolled thin in regular jewelers' rolls, and then annealed.

The ribbons are then cut into square pieces and put into a cutch made from ground parchment which holds about two hundred pieces. The cutch is then placed in parchment bands and beaten with a twenty-pound hammer for fifteen minutes on a granite block weighing about seven hundred pounds. Every five minutes the cutch is opened, and the gold examined; the cutch is then split in half and the position of the pieces reversed, so as to bring the center pieces on the outside and those on the outside into the center of the pack, the reason being that the gold in the center spreads more rapidly than that which comes next to the hammer. The reason of this we cannot explain, but simply state it as one of the facts concerning the manufacture of gold foil.

We continue beating this cutch, reversing it every five minutes, until the proper size is reached.

Four cutches are used in one beating, making eight hundred pieces of gold. The gold thus beaten is laid out of the cutch and filled into a mold made from the intestines of the ox, and as thin as the skins of this mold are, they are two stuck together. As there is a rough and a smooth side to each skin, the two rough sides are stuck together, making what is known as gold beaters' skin.

A mold contains eight hundred skins, which represent five hundred head of cattle. On the ends of each mold are fifteen or twenty old skins, called "wads," and outside of these several wads made from parchment. These wads protect the gold from the hammer. The mold before using, has to be put in order by pressing. This is done by putting it into a hot press between pasteboard, turning them every five minutes for one-half hour and then separating each skin to let out the dampness. This is called giving the mold a fly. Sometimes we have to give a mold two or three flies. It depends upon the weather. In winter when it is dry, the molds often need no pressing whatever. In fact, they are so dry the gold will sometimes stain. When the molds are too dry, they beat the gold too solid. At this time we look for complaints. The cry is, "Your gold works stiff; it rattles like a tin pan," etc. On the other hand in the summer when we have dog days, it tries the gold beaters' souls. We give the molds fly after fly and still they are not dry enough. The hammer then generates heat very rapidly, and we have to be very careful not to spoil our mold. This is done by generating so much heat by the hammer that the mold will be drawn in the center and absolutely ruined. At this time the gold will be so porous that if you hold it to the light it will look like a cobweb. At this time it works softer than it does when the moulds are dry, but the complaint is that it is not tough, the instrument cuts through, etc., so you will see that a gold beater's life is not all clear sailing.

When the cutch gold is filled into the mold, we place the mold with the eight hundred pieces of gold between the parchment bands the same as we did the cutch. We then beat on the mold about one hour with a ten-pound hammer, stopping every five minutes to examine the gold to see how it moves up. We have to be very coreful not to beat over the edges of the gold, if we do, the gold does not spread.

After the mold of eight hundred pieces is beaten to about four and one-half inches square, it is laid out, piece by piece, on a calf-skin cushion lined with flannel. It is then cut to size with what we call a wagon. This is a frame carrying two pieces of Malica reed, such as canes and musical instruments are made from, split into four pieces. When these reeds get dull, they are sharpened by taking a shaving off the edges. After the gold is cut to size, it is placed between pieces of paper. It is then taken and annealed on a piece of platina, piece by piece, to a cherry red. As unannealed gold is called for sometimes, it is put up without being annealed. Understand, I have been talking so far about Nos. 2, 3, 4, etc., of book foil. Later will give you the process of cylinder gold.

After the gold has been annealed, piece by piece, it is then weighed. A book is balanced in the scales, then one-eighth of an ounce put in and that is balanced. This gives one-eighth of an ounce to each book. It is then put into envelopes, marked, and is ready for sale.

In making gold for cylinders, it is beaten as described. It is then placed sheet by sheet, between paper and enclosed in an iron box, with weights on the gold. The iron box is then placed on a slow fire and allowed to smoulder, care being taken not to ignite the paper. After smouldering, the heat is gradually let on until the paper becomes carbon. As the paper shrinks, the gold shrinks with it. The carbon is then blown off, sheet by sheet, and we have what is called corrugated or crystalline gold. This is then rolled into form and cut to size, and made into style A. B. & C. cylinders. I have been told that corrugated gold was discovered at the time of the big fire in Chicago. When the gold was taken out of a safe in one of the dental depots, it was found to be corrugated. I claim to be the first one who made and sold corrugated gold; this was in 1867. Some years later a patent was taken out for the process and I was threatened with suit, unless I discontinued its manufacture. This I declined to do and no suit was entered.

I have now given you the process of refining and beating gold foil as practiced by me. I will now treat on non-cohesive gold, soft gold, cohesive gold, discoloration of gold, etc.

I have been amused many times in hearing dentists wrangle at conventions over the workings of non-cohesive gold, also to see it quoted, in many papers written by dentists for the journals, that non-cohesive gold contained impurities, and eminent chemists have assayed it from time to time and found a certain percent of impurities. Now doesn't it seem rather strange that no one as yet has ever been able to name the impurities? A chemist is supposed to be a pretty smart man; but facts are stubborn things, and one of these facts is that as yet no chemist has been able to name the so-called impurities in non-cohesive gold. I do not propose to tell how to make pure gold non-cohesive; in fact there are very few gold foil manufacturers that know how it is done. At present non-cohesive gold is used very little, and, as its use is not taught in most of the colleges of to-day, it looks as if its use would soon become one of the lost arts. However, if I am allowed my opinion, I wish to say that non-cohesive gold, used by one who understands it, and in cases where it is possible to use it, is one of the best filling materials to save teeth that we have. But how few there are who know how to use it. Those who do are passing away, and will soon join the silent majority.

Non-cohesive gold is called by some, unannealed. We make a strictly non-cohesive gold. At the present time the sale is very limited, and grows less every year. Those who use it are old practitioners; in fact, I do not know of any young dentist who uses it at all. I have heard some dentists at conventions speak of non-cohesive gold as unannealed gold. Now they would have a sorry time of using unannealed gold for wedge filling.

Some years ago the late Dr. Joshua Tucker, of Boston, came to me and ask me to explain, if I could, how it was that when he spoke at conventions about how difficult it was to use soft gold in some cases that some young student, just graduated, would jump up and tell how easy it was to use it. I told him it was easy to explain. "Well," he said, "I am writing a paper on gold, to leave behind, as I expect to stay on this earth but a short time, so, if you can give me any points on this working of gold, do so." I said to him, "When you used gold years ago, before you bought of us, you used Abby's soft gold." His answer was, "Yes." "Well, now," I said, "this gold was a non-cohesive gold. The gold the graduates of to-day are talking about is really a cohesive gold. In fact, it becomes cohesive by annealing, which the non-cohesive does not. In other words, with the graduates' soft gold you can build down teeth, but not so with Abby's soft gold, as that is, strictly speaking, a non-cohesive gold, and does not become cohesive by annealing." After this explanation to the old gentleman, he said, "Well, it is all plain to me now. I never could account for the fact that the young men of the profession could do so much much more with soft gold than I could."

Now, some who hear this will take exceptions to non-cohesive gold not being made cohesive by annealing. To those I will say, "Do not misunderstand me; I am talking about cohesion, not mechanical union or interlacing." Of course, it is possible to fill a tooth with non-cohesive gold and apparently make a cohesive filling of it, but the dentist who does this will find he has a delusion and a snare, because he has no cohesion. It is done by mechanical union and interlacing with sharp, serrating pluggers or packers, as one of you professors would say. Take this same non-cohesive gold in sheet, in fact, take any number of sheets, anneal them red hot, now place them on top of each other; now take the same number of sheets of cohesive gold and do the same, and note the result. The non-cohesive gold can be separated sheet by sheet, but the cohesive gold has cohered together in one mass. Now, by mechanical union force and sharp serrated pluggers, you can force the non-cohesive gold together. But is this cohesion ? We think not. In fact, you may be able to build down a tooth with non-cohesive gold by mechanical union and interlacing, but you have no strength. In fact, the filling is a delusion and a snare, and there is the trouble to-day with dentists who write for the dental journals. They do not know what they are talking about when they speak of cohesive and non-cohesive gold. They seem to think it is one and the same. Here is a late quotation from one of the journals: "If pure gold foil is exposed to the atmosphere for any length of time, it gathers upon its surface an imperceptible film, which, while not affecting the purity of the substance itself, it interferes with its cohesion. Two pieces of foil in this condition may be rubbed together without adhering, This is called non-cohesive." Now that is where a a great many dentists are led astray by such writings. Cohesive gold cannot be made non-cohesive any more than non-cohesive can be made cohesive. Any non-cohesive foil that can be made cohesive by annealing is not a non-cohesive foil. The more you anneal it red hot, the more non-cohesive it becomes. Speaking of cohesive and non-cohesive foil in one foil, is like saying a thing is very black and very white.

Some years ago when on a vacation, I called on a dentist and had several discussions on gold, finding him using non-cohesive gold. I told him I thought he must be mistaken on the cohesive point. The next day I called at his office, and he was engaged in building down a lateral incisor for a lady. He smiled on me, and says, " You see you are mistaken about non-cohesive gold, I am building down this tooth with it." I said nothing, but continued to watch him. He looked at me from time to time with a smile, childlike and bland, that said: You see you Boston folks don't know as much as you think you do. However, his smile soon changed to disgust. As he was giving it the finishing touches with the burnisher, the whole filling collapsed and dropped out. About that time I thought it was time to go to dinner and bid the Doctor "Good day." After dinner he called on me at the hotel and acknowledged that I was right, as he found, upon examination, that the filling could be picked apart, piece by piece, as it was put together. In fact, there was no cohesion about it. He had made the filling by mechanical union and interlacing.

Now then in regard to soft gold. I admit this is wrongly named. It should be semi-cohesive gold. This gold is made half way between the cohesive and non-cohesive. In other words, it is made the same as you would temper an instrument. We do not temper it so much as we do the non-cohesive. The way we do it we need these smart fellows to find out. Perhaps some chemist will tell you some day the means we use.

Cohesive gold is simply pure gold, as it comes from the refiner. It is not treated in any way. The great difficulty is to get it absolutely pure. You have no idea of the care and skill it takes to produce pure one-thousand-fine gold.

Cohesive gold has the peculiarity of welding cold, when it is freshly annealed and free from moisture or foreign matter. But how easy it is to destroy this cohesion. The fumes of most simple gases will do it. We have frequently had complaints of our gold working badly, when the trouble was that the painters had been to work painting the office; creosote, carbolic acid, etc., will also destroy the cohesion of gold. Then again how many destroy the good qualities of gold by overheating. I have seen some dentists melt gold in annealing it, and then complain that it works hard. Cohesive gold, if in the right condition, should be capable of cohering to itself without any pressure whatever. Anneal say six pieces more or less, place them on a table. Then take up one and gently drop it on the next piece, and then back to the next, and so on. If it is cohesive gold, you will find that you have all firmly welded together. Then, we have dentists say that our gold is too hard. Before we were acquainted with the workings of gold foil, this used to puzzle us—how pure gold could be hard. However, we soon found that the dentist did not know how to express himself. He really meant too cohesive and therefore welded together before he really got it where he wanted to put it. In fact the most cohesive gold ever made is as soft as non-cohesive. The difference is that one sticks together and the other does not. However, we admit that gold works differently at different times, caused by the condition of the skins, as explained in pressing the mold. These skins are like our hands. In summer they are moist, in winter dry. Therefore, foil does not require so much annealing in the winter as in the summer.

In regard to discoloration of gold filling in the mouth, I hardly feel that I have time to discuss this subject. It is one of the most annoying troubles that dentists have. As far as the gold is concerned, it cuts no figure. You will have to look for the cause elsewhere.

Some years ago we started in to find the cause, and have been on the road ever since, but have not found all the causes yet. However, here are many causes, and you take your choice. We have tried to make a gold that would discolor in the mouth, but failed. I had two dead upper bicuspid teeth. These were badly decayed at the margin of the gum. In fact, it was a good place to have filling discolor. I also had a lower tooth decayed. I prepared some gold for the two bicuspids alloyed with iron. This gold when annealed red hot turned as black as coal. When it was slightly annealed, it would not discolor, but was cohesive enough to make a good cohesive filling. These two bicuspids were built down with this gold. I took pains to keep away from these as much as possible when cleaning my teeth. However, these fillings have never shown any sign of discoloration. On the other hand, the lower tooth was filled with our best cohesive gold, and after about two years, it turned as black as coal, and is black to this day. This case was reported in the Johnston Bros.' Journal at the time by Professor Thomas Chandler. At this time I went through a long course of experiments with him, which are too numerous to mention. I also gathered together about one ounce of old discolored fillings, and took them to Dana Hayes, the State Assayer, who analyzed them for me and pronounced the black deposit sulphide of gold. These experiments were tried in 1875 and I satisfied myself at that time and have not had cause to change my mind since, that the discoloration of gold fillings is only on the surface and is no fault of the gold. I also found that by taking a piece of refined gold, say six inches long, burning one end in a little spirit lamp, a little globule would melt bright; burnish the other end, say two hundred times, more or less, and it would discolor a dark red, showing the gold took iron from the burnisher.

In conclusion I will show you the difference between the precious and base metals. I hold in my hand a bar of pure gold, also tin. You notice I can bend the tin back and forth with ease. In fact, after continued bending, the tin will fall apart. Now notice the difference with the pure gold. I bend it once, twice and that is all. It has stiffened up so it cannot be bent any more. Now this same thing takes place in a gold filling. The first blow you strike your foil causes it to stiffen. Therefore, be sure you get it placed before you strike the first blow. This holds good more in the precipitate gold than in the foil and cylinders. Place your gold into position before striking the first blow.

In conclusion, I will say I served my time as a gold beater and then was taught the art of refining gold and its manufacture into gold foil. I then traveled on the road, calling on all the prominent dentists in the country and large cities. I have stood over some of the best operators and witnessed a great many difficult operations, both in private offices and dental colleges, and must acknowledge that I owe much of my success as a gold-foil manufacturer to what I have gathered in this way.

